# Trends in the Surgical Treatment of Pseudotumor Cerebri Syndrome in the United States

**DOI:** 10.1001/jamanetworkopen.2020.29669

**Published:** 2020-12-15

**Authors:** Ali G. Hamedani, Dylan P. Thibault, Karen E. Revere, John Y. K. Lee, M. Sean Grady, Allison W. Willis, Grant T. Liu

**Affiliations:** 1 Department of Neurology, Perelman School of Medicine, University of Pennsylvania, Philadelphia; 2 Translational Center of Excellence for Neuroepidemiology and Neurology Outcomes Research, University of Pennsylvania, Philadelphia; 3 Department of Biostatistics, Epidemiology, and Informatics, Center for Clinical Epidemiology and Biostatistics, University of Pennsylvania, Philadelphia; 4 Department of Ophthalmology, Perelman School of Medicine, University of Pennsylvania, Philadelphia; 5 Division of Ophthalmology, Children’s Hospital of Philadelphia, Philadelphia, Pennsylvania; 6 Department of Neurosurgery, Perelman School of Medicine, University of Pennsylvania, Philadelphia; 7 Leonard Davis Institute of Health Economics, University of Pennsylvania, Philadelphia

## Abstract

**Importance:**

Optic nerve sheath fenestration (ONSF) and cerebrospinal fluid shunting are sometimes used to treat pseudotumor cerebri syndrome (PTCS), but their use patterns are unknown.

**Objectives:**

To investigate the frequency of surgical PTCS treatment in the United States and to compare patients undergoing ONSF with those treated with shunting.

**Design, Setting, and Participants:**

This was a retrospective longitudinal cross-sectional study. Inpatient data were obtained from the National Inpatient Sample (NIS), and outpatient surgical center data were obtained from the National Survey of Ambulatory Surgery (NSAS) and National Hospital Ambulatory Medical Care Survey (NHAMCS). Included in the analysis were 10 720 patients aged 18 to 65 years with a diagnosis code for PTCS, excluding venous thrombosis and other causes of intracranial hypertension. Time trends were explored and logistic regression was used to measure differences according to age, race/ethnicity, sex, Elixhauser comorbidity index, and other patient and hospital characteristics. Data analysis was performed from March 31 to October 7, 2020.

**Exposure:**

Treatment for PTCS, excluding venous thrombosis and other causes of intracranial hypertension.

**Main Outcomes and Measures:**

Annual number of PTCS-related admissions, ONSFs, and shunt procedures from 2002-2016. Patient and hospital-level characteristics of patients with PTCS undergoing ONSF or shunting were compared.

**Results:**

Between 2010 and 2016, 297 ONSFs were performed and 10 423 shunts were placed as treatment for PTCS. The procedures were most commonly performed in individuals aged 26 to 35 years (39.4%), and 9920 (92.4%) of the surgically treated patients were women. ONSF was more common among younger patients (eg, adjusted odds ratio [AOR] for patients ≥46 years vs those 18-25 years, 0.22; 95% CI, 0.08-0.61) and in Black, Hispanic, or other minority populations (AOR, 2.37; 95% CI, 1.31-4.30) and less common in the South (AOR, 0.34; 95% CI, 0.13-0.88) and West (AOR, 0.15; 95% CI, 0.04-0.58) compared with the Northeast. Total PTCS-related hospitalizations increased from 6081 (95% CI, 5137-7025) in 2002 to 18 020 (95% CI, 16 607-19 433) in 2016. Shunting increased from 2002 to 2011 and subsequently plateaued and declined. ONSF was used much less frequently, and use has not increased. No instances of outpatient ONSF or shunting for PTCS were recorded in the NSAS or NHAMCS databases.

**Conclusions and Relevance:**

This study’s findings suggest that shunting is more common than ONSF and that the use gap has widened as shunting has increased. However, because overall PTCS-related hospitalizations have increased even more rapidly, the percentage of inpatients with PTCS undergoing surgery has decreased. These trends may reflect changes in medical treatment practices and outcomes or growing limitations in access to ophthalmic surgical expertise.

## Key Points

**Question:**

How often is optic nerve sheath fenestration (ONSF) used relative to cerebrospinal fluid shunting procedures to treat pseudotumor cerebri syndrome (PTCS) in the United States?

**Findings:**

In a cross-sectional analysis of nationally representative hospitalization data on 10 720 surgical procedures from the National Inpatient Sample, shunting was more than 10 times more common than ONSF, and this gap has widened as shunting for PTCS increased from 2002 to 2016. However, because the overall rate of PTCS-related hospitalization has increased even more rapidly, the percentage of inpatients with PTCS undergoing surgery has decreased.

**Meaning:**

This study’s findings suggest that ONSF is used less frequently than shunting for PTCS, which may reflect underlying trends in medical treatment practices and outcomes or growing limitations in access to ophthalmic surgical expertise.

## Teaser

This cross-sectional analysis of nationally representative hospitalizations uses data from the National Inpatient Sample and 3 national surveys of ambulatory surgery to compare the frequency of use of optic nerve sheath fenestration vs cerebrospinal fluid shunt placement for the treatment of pseudotumor cerebri syndrome.

## Introduction

Pseudotumor cerebri syndrome (PTCS), also known as idiopathic intracranial hypertension, is a syndrome of increased intracranial pressure in the absence of a mass lesion, hydrocephalus, or underlying infection or inflammation; it is often associated with weight gain, especially in young women of childbearing age.^1^ Optic nerve swelling (papilledema) is the clinical hallmark of PTCS and can lead to irreversible vision loss if untreated. Headache, transient visual obscurations, double vision, and pulsatile tinnitus are also common symptoms. The majority of patients with PTCS are effectively treated with oral acetazolamide and weight loss. However, surgical treatment with optic nerve sheath fenestration (ONSF) or cerebrospinal fluid (CSF) shunting is sometimes performed for patients with severe or refractory cases.^2^ In an ONSF, a temporary surgical opening in the dural sheath that surrounds the optic nerve is created, thereby releasing the local pressure responsible for papilledema and vision loss. In a shunting procedure, an indwelling catheter is placed to provide continuous drainage of cerebrospinal fluid from the lateral ventricle or lumbar subarachnoid space to the peritoneal cavity, which lowers intracranial pressure and relieves headache, papilledema, and other symptoms.

Although ONSF and shunting procedures have been performed for decades, their current patterns of use in the treatment of PTCS are unknown. In large academic centers, up to 10% of patients with PTCS are treated with surgery,^3^ but treatment patterns likely differ in the general population. Surveillance data from 1988 to 2002 suggest that shunting was performed more frequently (and its use was increasing more rapidly) than ONSF,^4^ but subsequent trends over the past decade are unknown. Aside from individual patient characteristics and provider preferences, we do not know whether population-level factors also influence the choice of ONSF or shunting as surgical treatment for an individual with PTCS.

Understanding the trends and determinants of use of surgical procedures in the treatment of PTCS is important for several reasons. First, treatment patterns can provide clues to underlying changes in disease epidemiology and treatment outcomes at a systemwide level. For example, if surgical treatment for PTCS is decreasing, it could indicate that medical treatment is being more widely and effectively implemented and that the burden of severe vision loss due to PTCS is decreasing. In addition, racial and socioeconomic health disparities, which affect every field of medicine, have not been examined in the context of PTCS, and ensuring that care is equally available to all who need it is a public health priority. Knowledge of current treatment patterns provides critical context in terms of how future clinical research results will impact clinical care. The comparative effectiveness of ONSF and shunting for PTCS is currently unknown: evidence is limited to uncontrolled single-center case series, and a randomized clinical trial of medication plus surgery compared with medication alone was terminated early because of low enrollment. However, if one surgical procedure is ultimately found to be superior to the other, the extent to which clinical practice will change (and whether the health care community is prepared for this change) depends on how frequently the 2 procedures are being performed.

To explore recent national trends in the surgical treatment of PTCS, we investigated the annual frequency of ONSF, shunting, and hospitalization for treatment of PTCS in the United States and compared the clinical and demographic characteristics of patients undergoing ONSF or shunting using the National Inpatient Sample (NIS), a national database of hospital discharge data, and 3 national surveys of ambulatory surgery. We hypothesized that shunting would be more common than ONSF but that relative differences in surgical frequency would be stable over time.

## Methods

This study was exempt from approval by the University of Pennsylvania Institutional Review Board because of the fully deidentified nature of the data set, and therefore, informed consent was not required. Reporting adhered to the Strengthening the Reporting of Observational Studies in Epidemiology (STROBE) reporting guideline for cross-sectional studies.^5^

To examine inpatient surgical procedures for PTCS, we used data from the NIS, which is the largest all-payer inpatient health care database in the United States and is made available through the Healthcare Cost and Utilization Project (HCUP) by the Agency for Healthcare Research and Quality.^6^ Before 2012, the NIS included all discharges from a 20% stratified sample of US hospitals. Since 2012, the NIS has contained a 20% stratified sample (>7 million hospitalizations annually) of discharges from all US hospitals. To achieve a stratified sample of hospitalizations, HCUP first divides all eligible hospitals into strata according to census division, location, size, teaching status, and ownership. A systematic 20% random sample of hospitalizations is then taken from each hospital, and sample weights are assigned to each hospitalization according the proportion of total hospitalizations that are represented within each stratum. This accounts for clustering within hospitals and ensures that no single hospital skews the results of any weighted analyses. The database contains deidentified encounter-level information on patient and hospital demographic characteristics, diagnoses and comorbidities, inpatient procedures, and health care costs and payer information. Diagnoses and procedures are recorded using *International Classification of Diseases, Ninth Revision, Clinical Modification* (*ICD-9-CM*) codes (for hospitalizations prior to October 1, 2015) and *International Classification of Diseases, Tenth Revision, Clinical Modification* (*ICD-10-CM*) codes (for hospitalizations after October 1, 2015). For our analysis, we analyzed data from the years 2002-2016. We selected 2002 as the starting point for our trends analysis to maintain continuity with a previous analysis of shunting in PTCS using NIS data from 1998-2002,^4^ and 2016 was the most recently available year of NIS data at the time of analysis.

To examine outpatient surgical procedures for PTCS, we used the 2006 National Survey of Ambulatory Surgery (NSAS)^7^ and the 2010 and 2011 National Hospital Ambulatory Medical Care Survey (NHAMCS).^8^ NSAS and NHAMCS are national health care surveys conducted by the US Centers for Disease Control and Prevention’s National Center for Health Statistics (NCHS). Together, they contain data on a nationally representative stratified sample of more than 119 000 ambulatory surgical procedures performed in hospital-based and freestanding ambulatory surgical centers along with detailed diagnostic and procedural information. The NSAS was only conducted once in 2006; NHAMCS has been conducted annually from 1992 and 2017, but we limited our analysis to 2010 and 2011, because these are the only 2 years in which it contained both outpatient hospital and ambulatory surgical center procedures.

The NIS data are publicly available for purchase through HCUP, and NSAS and NHAMCS data are publicly available at no cost through NCHS.

### Population

We identified all health care encounters with PTCS using *ICD-9-CM* code 348.2 and *ICD-10-CM* code G93.2 in either the primary or secondary diagnosis position. Consistent with established diagnostic criteria for PTCS,^1^ we excluded secondary causes of papilledema, such as cerebral venous thrombosis, hydrocephalus, meningitis or encephalitis, and brain tumor, abscess, or other intracranial mass lesions. We then used *ICD-9-CM* and *ICD-10-CM* procedural codes (primary or secondary position) to identify ONSF and shunting procedures (which includes both ventriculoperitoneal and lumboperitoneal shunts) among patients with PTCS (eTable 1 in the Supplement). These codes identify first-time surgical interventions and are different from the codes that identify surgical revisions or a history of surgery from a previous visit. However, the timing of ONSF or shunt placement within a given hospitalization cannot be determined. Although venous sinus stenting has recently emerged as a surgical alternative for treatment of PTCS, we were unable to examine this in NIS because of the absence of unique *ICD-9-CM* or *ICD-10-CM* codes that can reliably identify this procedure in administrative health care data sets. We restricted the study population to individuals aged 18 to 65 years. A flowchart of the inclusion and exclusion criteria, as applied to our cross-sectional analyses, is shown in the eFigure in the Supplement.

In addition to producing national estimates of frequency of use of ONSF and shunting for PTCS, we examined whether PTCS surgery type was associated with patient characteristics, such as age group, sex, insurance provider (Medicare, Medicaid, private, self-pay, or no charge/other), and median income by zip code (≤$37 999, $38 000-$47 999, $48 000-$63 999, and ≥$64 000), as well as hospital-level factors, such as size (small, medium, or large as defined by HCUP), teaching status, and geographic region (Northeast, South, Midwest, or West). We also examined race/ethnicity (White, Black, Hispanic, Asian or Pacific Islander, Native American, or other), which is reported by HCUP based on hospital documentation because of known disparities in health care access and PTCS-related outcomes.^9^ Severity of comorbid medical illness was quantified using the Elixhauser comorbidity index.^10^ For these analyses, we excluded records with missing data for key variables (eFigure in the Supplement).

### Statistical Analysis

The data analysis was performed from March 31 to October 7, 2020. We tabulated the number of ONSF and shunt procedures for PTCS as well as the number of total hospital admissions for treatment of PTCS by calendar year from 2002-2016. To capture more recent health care use patterns, we used NIS data from 2010-2016 to summarize the patient and hospital-level characteristics of patients with PTCS undergoing ONSF or shunting and compared them using single-variable and multivariable logistic regression (adjusted for age, race/ethnicity, sex, Elixhauser comorbidity index, median income by zip code, insurance payer, hospital size, hospital location, and hospital teaching status). We also included an *ICD-10-CM* vs *ICD-9-CM* indicator variable in multivariable regression models because convoluted *ICD-9-CM* to *ICD-10-CM* conversion ratios can cause spurious increases or decreases in diagnosis or procedure frequency across years.^11^ When both ONSF and shunt placement occurred during the same admission (n = 35 from 2010-2016), it was included in descriptive statistics but excluded from regression models. Statistical analyses were performed using SAS software, version 9.4 (SAS Institute Inc). To account for the sampling design of the NIS and ensure accurate estimates and appropriate CIs for both descriptive statistics and logistic regression models, we used survey analytic methods (PROC SURVEYMEANS and PROC SURVEYLOGISTIC) with Taylor series variance estimation and HCUP-defined variables for stratum (NIS_STRATUM) and primary sampling unit (HOSP_NIS). We used modified trend weights for the years 2002-2011 to account for the redesign of the NIS sampling frame in 2012 as recommended by HCUP.^12^ Statistical significance was defined as *P* < .05; all tests were 2-sided.

## Results

Between 2010 and 2016, 10 720 surgical procedures were performed for patients with PTCS: 297 ONSFs and 10 423 shunts. The clinical and demographic characteristics of patients with PTCS undergoing ONSF or shunting from 2010-2016 are shown in the Table.^13,14,15,16^ These surgical procedures were most commonly performed for patients aged 26 to 35 years (39.4%), and 9910 (92.4%) of the surgically treated patients were women, consistent with the epidemiology of PTCS. Compared with patients aged 18 to 25 years, those in older age groups were more likely to receive a shunt than ONSF according to both unadjusted and adjusted analyses (eg, adjusted odds ratio [AOR] for patients aged ≥46 years vs those 18-25 years, 0.22; 95% CI, 0.08-0.61). Black, Hispanic, or other minority populations were more than twice as likely to receive ONSF as a shunt (AOR, 2.37; 95% CI, 1.31-4.30). Compared with the Northeast, ONSF was used less frequently than shunting in the South (AOR, 0.34; 95% CI, 0.13-0.88) and West (AOR, 0.15; 95% CI, 0.04-0.58). No differences between ONSF and shunting were observed according to sex, medical comorbidities, other sociodemographic factors (median household income, insurance payer), or hospital-level factors (size, teaching status).

**Table.** Patient and Hospital-Level Factors Associated With a Greater Likelihood of Optic Nerve Sheath Fenestration Rather Than Cerebrospinal Fluid Shunting for Pseudotumor Cerebri Syndrome, National Inpatient Sample 2010-2016

Factor	No. (%)^a^		OR (95% CI)	
	Optic nerve sheath fenestration (n = 297)	Cerebrospinal fluid shunt (n = 10 423)	Unadjusted (N = 10 720)	Adjusted (N = 10 720)^b^
**Patients**				
Age, y				
18-25	113 (5.5)	1953 (94.5)	1 [Reference]	1 [Reference]
26-35	109 (2.6)	4117 (97.4)	0.46 (0.26-0.81)	0.48 (0.27-0.87)
36-45	55 (1.9)	2916 (98.1)	0.33 (0.16-0.66)	0.33 (0.16-0.68)
≥46	20 (1.4)	1437 (98.6)	0.24 (0.09-0.66)	0.22 (0.08-0.61)
Race/ethnicity^c^				
White	140 (2.0)	6990 (98.0)	1 [Reference]	1 [Reference]
Black, Hispanic, Asian or Pacific Islander, and Native American	133 (5.0)	2545 (95.0)	2.61 (1.47-4.65)	2.37 (1.31-4.30)
Other or unknown	25 (2.7)	888 (97.3)	1.41 (0.53-3.74)	1.57 (0.54-4.56)
Sex				
Male	20 (2.5)	790 (97.5)	1 [Reference]	1 [Reference]
Female	277 (2.8)	9633 (97.2)	1.13 (0.43-2.97)	1.1 (0.40-3.04)
Insurance payer				
Medicare	20 (1.3)	1488 (98.7)	0.48 (0.17-1.36)	0.5 (0.17-1.43)
Medicaid	89 (3.2)	2709 (96.8)	1.18 (0.66-2.14)	0.82 (0.44-1.53)
Private	149 (2.7)	5396 (97.3)	1 [Reference]	1 [Reference]
Self-pay	25 (6.8)	341 (93.1)	2.62 (0.96-7.15)	2.2 (0.78-6.15)
Other	15 (3.0)	489 (97.0)	1.08 (0.33-3.46)	1.16 (0.34-3.94)
Median income by zip code, percentile^d^				
0-25th	123 (3.8)	3104 (96.2)	2.23 (0.94-5.30)	1.62 (0.62-4.22)
26th-50th	85 (2.7)	3091 (97.3)	1.54 (0.59-4.03)	1.43 (0.52-3.92)
51st-75th	60 (2.3)	2568 (97.7)	1.31 (0.49-3.49)	1.16 (0.43-3.14)
76th-100th	30 (1.8)	1659 (98.2)	1 [Reference]	1 [Reference]
Elixhauser comorbidity index^e^				
0	58 (2.2)	2579 (97.8)	1 [Reference]	1 [Reference]
1	90 (3.1)	2823 (96.9)	1.41 (0.64-3.13)	1.45 (0.65-3.23)
2	60 (2.5)	2327 (97.5)	1.14 (0.52-2.5)	1.34 (0.59-3.05)
≥3	89 (3.2)	2693 (96.8)	1.46 (0.70-3.02)	2.03 (0.96-4.30)
**Hospitals**				
Hospital size^f^				
Small	15 (2.6)	562 (97.4)	1 [Reference]	1 [Reference]
Medium	64 (3.8)	1625 (96.2)	1.48 (0.39-5.62)	1.52 (0.38-6.05)
Large	218 (2.6)	8236 (97.4)	0.99 (0.32-3.10)	1.3 (0.40-4.24)
Hospital teaching status				
Rural^g^	<10	222	0.66 (0.10-4.49)	0.93 (0.13-6.87)
Urban				
Nonteaching	25 (1.7)	1458 (98.3)	0.56 (0.13-2.39)	0.85 (0.19-3.81)
Teaching	268 (3.0)	8742 (97.0)	1 [Reference]	1 [Reference]
Region				
Northeast	73 (4.6)	1523 (95.4)	1 [Reference]	1 [Reference]
South	45 (1.9)	2373 (98.1)	0.39 (0.15-0.99)	0.34 (0.13-0.88)
Midwest	164 (3.6)	4429 (96.4)	0.77 (0.36-1.66)	0.66 (0.30-1.45)
West	15 (0.7)	2098 (99.3)	0.15 (0.04-0.55)	0.15 (0.04-0.58)

Abbreviations: HCUP, Healthcare Cost and Utilization Project; OR, odds ratio.

^a^ Row percentages for the first 2 columns are shown in parentheses.

^b^ Adjusted for age, race/ethnicity, sex, insurance payer, median income by zip code, Elixhauser comorbidity index, hospital size, teaching status, region, and an *International Classification of Diseases, Tenth Revision, Clinical Modification* vs *International Classification of Diseases, Ninth Revision, Clinical Modification* dummy variable (to account for the coding transition in 2015).

^c^ Race: The categories for race/ethnicity reflect the options provided for HCUP coding of data elements.^13^

^d^ Income: Descriptions of income quartiles are available from the HCUP website.^14^

^e^ Scores are defined by Elixhauser et al.^15^

^f^ Hospital size categories are defined by the HCUP according to teaching status and geographic location.^16^

^g^ Counts and percentages are not presented for cells with less than 10 individuals to protect patient confidentiality per HCUP regulations.

**Figure 1.**
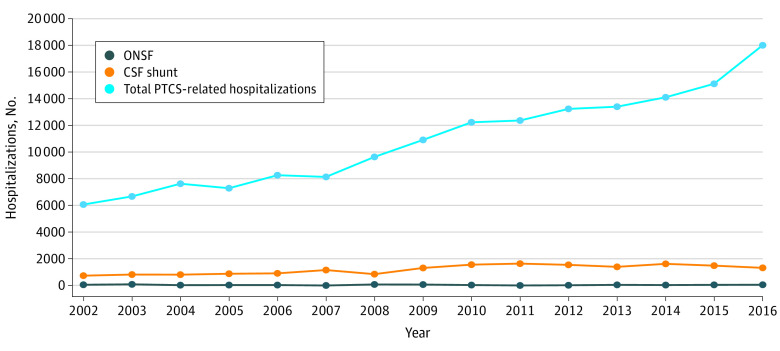
Number of Optic Nerve Sheath Fenestrations (ONSF), Cerebrospinal Fluid (CSF) Shunting Procedures, and Total Hospitalizations for Pseudotumor Cerebri Syndrome (PTCS), National Inpatient Sample, 2002-2016

The total number of PTCS-related hospitalizations and the subset during which ONSF or shunting was performed are shown in Figure 1. In 2002, an estimated 6081 (95% CI, 5137-7025) hospitalizations of patients with a diagnosis of PTCS occurred in the United States, and this number has increased consistently over time such that 18 020 (95% CI, 16 607-19 433) PTCS-related hospitalizations were reported in 2016. This 196% increase far exceeds the 12% increase in the total US population between the ages of 20 and 64 years according to the US Census Bureau’s annual American Community Survey.^17^ The largest increase in hospitalizations for treatment of PTCS occurred between 2015 and 2016, when there were 2890 more hospitalizations than in the preceding year. A closer inspection of the data reveals that much of this increase occurred between the third and fourth quarters of 2015, which is when the United States switched from the *ICD-9-CM* coding system to the *ICD-10-CM* coding system (eTable 2 in the Supplement). Similar discrepancies in disease prevalence between *ICD-9-CM* and *ICD-10-CM* coding definitions have been previously described for other neurologic and non-neurologic conditions.^11^ No instances of outpatient ONSF or shunting for PTCS were recorded in the 2006 NSAS or 2010 and 2011 NHAMCS databases.

**Figure 2.**
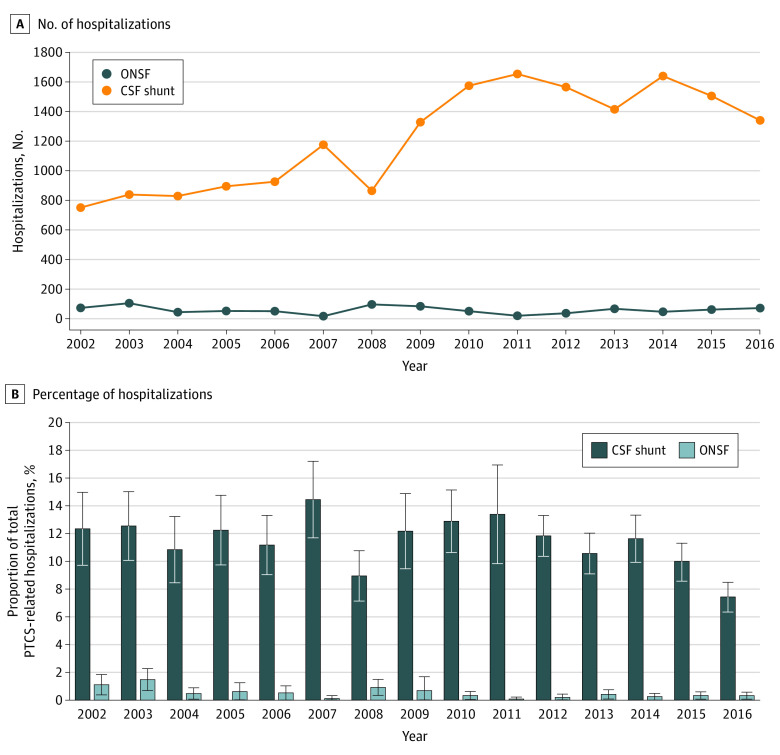
Number of Optic Nerve Sheath Fenestrations (ONSF) and Cerebrospinal Fluid (CSF) Shunting Procedures for Pseudotumor Cerebri Syndrome (PTCS), National Inpatient Sample, 2002-2016 Percentages indicate the proportion of total PTCS-related hospitalizations accompanied by each procedure. Whiskers indicate 95% CI.

The proportion of hospital admissions for treatment of PTCS accompanied by either ONSF or a shunt procedure was low. In 2002, 750 of 6081 PTCS-related hospitalizations included shunt placement (12.3%; 95% CI, 9.7%-15.0%), and only 71 (1.2%; 95% CI, 0.5%-1.9%) included ONSF. Shunting was thus used approximately 10 times more frequently than ONSF. Compared with the increasing number of total hospitalizations for treatment of PTCS, the annual number of ONSF or shunt procedures in Figure 1 appears relatively stable over time. However, after removing the total number of admissions for treatment of PTCS and viewing the same surgical data on a smaller scale (Figure 2), it becomes apparent that the number of shunt procedures increased between 2002 and 2016, whereas the frequency of ONSF remained unchanged. The greatest increase in shunt prevalence occurred between 2008 and 2011, peaking at 1654 shunt placement procedures (95% CI, 1094-2215) in 2011, followed by a gradual decrease. Thus, 10.6 shunts were placed for every ONSF in 2002, but this ratio increased to 36.4:1 by 2016. However, because the rate of increase in total admissions for treatment of PTCS was greater than that of shunts or ONSF, the percentage of PTCS-related hospitalizations accompanied by a surgical procedure decreased. In 2016, the proportion of PTCS-related hospitalizations accompanied by shunting or ONSF was 7.4% (95% CI, 6.4%-8.5%) and 0.4% (95% CI, 0.1%-0.6%), respectively, down from 12.3% (95% CI, 9.7%-15.0%) and 1.2% in 2002 (95% CI, 0.5%-1.9%).

## Discussion

In this cross-sectional analysis of 15 years of national hospitalization data in the United States, we found that the frequency of PTCS-related hospitalization has increased from approximately 6000 hospitalizations per year in 2002 to more than 18 000 hospitalizations in 2016. Shunting for PTCS has also increased, albeit to a lesser degree, and ONSF is used much less frequently, and use has not changed over time. We also uncovered age, racial, and geographic differences in the use of ONSF vs shunting for treatment of PTCS. These trends may reflect changes in medical practice patterns and treatment outcomes or growing limitations in access to ophthalmic surgical expertise.

Our findings suggest that PTCS diagnoses in the United States are increasing. This is consistent with previous county-level data and has been linked to the increasing prevalence of obesity, the primary risk factor for PTCS.^18^ However, because PTCS is still relatively rare (incidence of 22 per 100 000 individuals in high-risk groups), longitudinal trends are difficult to capture within a single catchment area and are more clearly illustrated here using national data. Of note, because NIS is an inpatient database and most patients with PTCS are diagnosed and treated as outpatients, we were unable to estimate the actual prevalence of PTCS in the United States. In addition, NIS is an encounter-level rather than a patient-level database; thus, it is possible that the same patient was admitted and counted more than once. However, unless readmissions for treatment of PTCS are increasing (for which there is no evidence at this time), the longitudinal trends in NIS data likely reflect an actual increase in disease prevalence.

We also found that the frequency of shunting for PTCS increased from 2002 to 2011 but has subsequently stabilized and trended downward. The initial increase from 2002 to 2011 mirrors previous trends in national inpatient data from 1988 to 2002.^4^ However, in previous studies, changes in the total prevalence of PTCS have not been considered. Although the frequency of both shunting and total PTCS-related hospitalizations has increased, we found that the rate of increase in PTCS-related hospitalizations was greater than that of shunting (as indicated by the slopes of the lines in Figure 1 and the decreasing percentages in Figure 2). This indicates that the relative use of shunt procedures for PTCS is decreasing; that is, fewer PTCS-related hospitalizations are accompanied by shunt placement now than in previous years. The reasons for the recent decline in shunting for PTCS are unclear. Given that the timing roughly coincides with the execution and dissemination of the results of the Idiopathic Intracranial Hypertension Treatment Trial^19^ (which provided conclusive evidence for the efficacy of acetazolamide in the treatment of PTCS), it is possible that early diagnosis and prompt initiation of medical treatment have become more widespread, reducing the need for surgical intervention. It is also possible that shunting is gradually being replaced by venous sinus stenting; we were unable to confirm this because of the lack of validated procedure codes for venous sinus stenting in PTCS. However, although stenting has increased in recent years, the literature remains limited to case series from a handful of centers^20^; therefore, we do not believe that this is sufficient to explain the magnitude of decline in shunting that we observed in the NIS data. Of note, the plateau in the rate of shunting for PTCS after 2011 also coincides with the redesign of the NIS sampling frame in 2012. However, we used appropriate sampling weights for trends analysis,^12^ and a change in sampling methods would, at most, cause a one-time shift in procedural counts rather than a change in longitudinal trend, so we do not think that this accounts for our findings.

Compared with shunts, ONSF is used much less frequently, and its use has not increased over time; in 2016, shunt placement was more than 36 times more common than ONSF. A similar pattern has been observed in administrative data from the United Kingdom.^21^ The reasons for the striking discrepancy between ONSF and shunt placement are unclear but may be related to the availability of surgical expertise. ONSF is performed primarily by ophthalmologists with subspecialty training in orbital and oculoplastic surgery,^22^ who are in relatively limited supply. (The American Society of Ophthalmic Plastic Surgery and Reconstructive Surgery currently has 650 practicing members in the US.^23^) In contrast, shunt placement is a standard skill developed in neurosurgical training and is widely performed by neurosurgeons, of whom there are more than 3600 in the US.^24^ Recent technological advances in electromagnetic navigation have also increased the surgical success of ventriculoperitoneal shunting in the treatment of PTCS.^25^ Although the comparative effectiveness of these 2 surgical procedures in the treatment of PTCS remains unknown, knowing that shunting is far more frequently used than ONSF has important implications for systemwide responses to clinical research results. For example, if ONSF were found to be superior to shunting in the future, a sudden increase in the demand for ONSF would likely require an increase in the number of surgeons who are trained to perform this procedure.

Because ONSF and shunting have different surgical indications (with the former providing temporary relief of vision loss and the latter able to provide sustained relief of headache and other symptoms), it is also possible that a shift in surgical preference reflects an underlying change in the clinical epidemiology and medical treatment outcomes of PTCS. For example, improved recognition, diagnosis, and medical treatment may have reduced the population-wide burden of early vision loss due to PTCS, thus limiting the need for ONSF. Of note, because NIS is an inpatient health care database, it does not contain data on outpatient surgical procedures performed in hospitals or ambulatory surgical centers unless they result in postoperative hospital admission. Although virtually all patients undergoing nonspinal neurosurgical procedures are admitted overnight for postoperative monitoring, many patients undergoing ophthalmic procedures (including essentially all corneal and cataract, glaucoma, and vitreoretinal surgery) have surgery performed in the outpatient setting; therefore, the data are not captured in an inpatient health care database such as NIS. The small number of NIS records with ONSF procedural codes also limits the reliability of weighted estimates, resulting in relatively wide CIs. Because the estimated number of ONSF procedures in NIS was lower than expected, it is likely that at least some of these procedures were performed in the ambulatory setting and that the total number of ONSFs performed annually for PTCS was higher. However, the fact that there were no instances of outpatient ONSF for PTCS in 3 different Centers for Disease Control and Prevention databases suggests that outpatient ONSF is relatively uncommon, and even if an equal number of inpatient and outpatient ONSFs were being performed for PTCS, ONSFs would still be eclipsed by shunting.

We also found that ONSF was more likely to be performed in younger patients and those from racial and ethnic minority backgrounds and was less frequently used in the South and West compared with the Northeast. Some of these differences may be associated with variability in the clinical presentation of PTCS and differing surgical indications. In an ONSF, an opening is created in the dural sheath surrounding the optic nerve, allowing cerebrospinal fluid to be released and the pressure around the optic nerve to be lowered. However, this opening may be temporary and eventually close spontaneously; therefore, the benefit of ONSF might be inherently self-limited. In contrast, an indwelling shunt can remain indefinitely and is therefore more useful than ONSF in patients with refractory cases when multiple attempts at nonsurgical treatment have already been unsuccessful. There is evidence that Black patients with PTCS present with more severe papilledema and vision loss and are therefore more likely to be treated with ONSF.^9^ Although shunts are generally safe, there are long-term risks of shunt malfunction requiring surgical revision, suggesting that this may be why they are less frequently used in younger patients who face a longer period with the shunt in place. We did not observe any differences according to insurance payer, and, to our knowledge, both procedures are covered equally by health insurers.

### Limitations

One important limitation of this study is its reliance on administrative claims coding of PTCS. The misdiagnosis of PTCS in clinical practice is well recognized,^26^ and, in validation studies, a single *ICD-9-CM* code for PTCS has a positive predictive value of only 55% to 65% for identifying PTCS. However, this increases to nearly 90% when an acetazolamide prescription is required.^27^ The NIS does not contain medication information, but by restricting our primary analysis to patients with PTCS who were treated with ONSF or a shunt, we believe that this similarly increases diagnostic validity in this study. Another limitation is the lack of patient-level information regarding severity of papilledema and vision loss, time from diagnosis to surgery, and treatment efficacy, which will be the focus of future studies. We were also unable to include venous sinus stenting because of methodological limitations, which makes it difficult to assess whether longitudinal trends in the use of ONSF or shunting are offset by a rise in this third surgical procedure.

## Conclusions

In summary, this cross-sectional study presents novel population-level data on national trends in the prevalence and surgical treatment of PTCS. An overall decline in the relative use of surgery may indicate that early diagnosis and prompt initiation of medical treatment have become more widespread, reducing the need for surgical intervention. Given the large discrepancy between ONSF and shunting, future large-scale comparative effectiveness studies may be limited by a small number of ONSFs, and a finding in favor of ONSF would require a large shift in health care utilization.

## Notes

Supplement. **eTable 1.** Diagnostic and Procedural Code Definitions for Optic Nerve Sheath Fenestration, CSF Shunting, and Exclusionary Diagnoses **eTable 2.** Number of ONSF and CSF Shunt Procedures for PTCS by Quarter, NIS 2015-2016 **eFigure.** Flowchart of Inclusion and Exclusion Criteria for Analyses of ONSF vs. CSF Shunting for PTCS, NIS 2010-2016
