# Supplementary material for: Trends in the Surgical Treatment of Pseudotumor Cerebri Syndrome in the United States
Source: JAMA Netw Open. 2020 Dec 15;3(12):e2029669. doi: 10.1001/jamanetworkopen.2020.29669 (PMC7739135; doi:10.1001/jamanetworkopen.2020.29669)
Supplement: Supplement. — eTable 1. Diagnostic and Procedural Code Definitions for Optic Nerve Sheath Fenestration, CSF Shunting, and Exclusionary Diagnoses eTable 2. Number of ONSF and CSF Shunt Procedures for PTCS by Quarter, NIS 2015-2016 eFigure. Flowchart of Inclusion and Exclusion Criteria for Analyses of ONSF vs. CSF Shunting for PTCS, NIS 2010-2016 [file jamanetwopen-e2029669-s001.pdf]

Supplementary Online Content

Hamedani AG, Thibault DP, Revere KE, et al. Trends in the surgical treatment of pseudotumor cerebri syndrome in the United States. *JAMA Netw Open*. 2020;3(12):e2029669. doi:10.1001/jamanetworkopen.2020.29669

eTable 1. Diagnostic and Procedural Code Definitions for Optic Nerve Sheath Fenestration, CSF Shunting, and Exclusionary Diagnoses

eTable 2. Number of ONSF and CSF Shunt Procedures for PTCS by Quarter, NIS 2015-2016

eFigure. Flowchart of Inclusion and Exclusion Criteria for Analyses of ONSF vs. CSF Shunting for PTCS, NIS 2010-2016

This supplementary material has been provided by the authors to give readers additional information about their work.

©2020 Hamedani AG et al. *JAMA Network Open.*

eTable 1. Diagnostic and procedural code definitions for optic nerve sheath fenestration, CSF shunting, and exclusionary diagnoses

Diagnosis or procedure	ICD-9 code(s)	ICD-10 code(s)
Optic nerve sheath fenestration	04.42 04.03 04.04	009G00Z 009G0ZZ 009G3ZZ 009G40Z 009G4ZZ 00CG0ZZ 00CG3ZZ 00CG4ZZ 00NG0ZZ 00NG3ZZ 00NG4ZZ 008G0ZZ 008G3ZZ 008G4ZZ
CSF shunting	02.31 02.32 02.33 02.34 02.35 02.39 02.42 02.43 03.71 03.72 03.79	0016070 0016071 0016072 0016073 0016074 0016075 0016076 0016077 0016078 001607B 00160J0 00160J1 00160J2 00160J3 00160J4 00160J5 00160J6 00160J7 00160J8 00160JB 00160K0 00160K1 00160K2 00160K3 00160K4 00160K5 00160K6 00160K7 00160K8 00160KB 00160ZB 0016370 0016371 0016372 0016373 0016374 0016375 0016376 0016377 0016378 001637B 00163J0 00163J1 00163J2 00163J3 00163J4 00163J5 00163J6 00163J7 00163J8 00163JB 00163K0 00163K1 00163K2 00163K3 00163K4 00163K5 00163K6 00163K7 00163K8 00163KB 00163ZB 0016470 0016471 0016472 0016473 0016474 0016475 0016476 0016477 0016478 001647B 00164J0 00164J1 00164J2 00164J3 00164J4 00164J5 00164J6 00164J7 00164J8 00164JB 00164K0 00164K1 00164K2 00164K3 00164K4 00164K5 00164K6 00164K7 00164K8 00164KB 00164ZB 001U072 001U074 001U076 001U077 001U079 001U0J2 001U0J4 001U0J6 001U0J7 001U0J9 001U0K2 001U0K4 001U0K6 001U0K7 001U0K9 001U372 001U374 001U376 001U377 001U379 001U3J2 001U3J4 001U3J6 001U3J7 001U3J9 001U3K2 001U3K4 001U3K6 001U3K7 001U3K9 001U472 001U474 001U476 001U477 001U479 001U4J2 001U4J4 001U4J6 001U4J7 001U4J9 001U4K2 001U4K4 001U4K6 001U4K7 001U4K9 0W110J9 0W110JB 0W110JG 0W110JJ
Cerebral venous thrombosis	437.6	I67.6
Hydrocephalus	331.3 331.4 331.5	G91.0 G91.1 G91.3 G91.8 G91.9
Meningitis/encephalitis	HCUP Clinical Classification Software 76 and 77	
Brain tumor, abscess, etc.	191.0 191.1 191.2 191.3 191.4 191.5 191.6 191.7 191.8 191.9 192.0 192.1 192.2 192.3 192.8 192.9 198.3 198.4 225.0 225.1 225.2	C70xx C71xx C72xx C793-C794xx D32xx D33xx D4xx G93.0 G93.5 G93.6, G96.19 G06xx G07xx

©2020 Hamedani AG et al. *JAMA Network Open.*

	225.3 225.4 225.8 225.9 237.5 237.6 239.6 239.7 348.0 348.4 348.5 349.2 324.0 324.1 324.9	

©2020 Hamedani AG et al. *JAMA Network Open.*

eTable 2. Number of ONSF and CSF shunt procedures for PTCS by quarter, NIS 2015-2016

	2015 q1	2015 q2	2015 q3	2015 q4	2016 q1	2016 q2	2016 q3	2016 q4
Total PTCS admissions	3700 (3327-4073)	3310 (2947-3673)	3765 (3385-4145)	4345 (3929-4761)	4385 (3964-4806)	4295 (3897-4693)	4915 (4474-5356)	4415 (4003-4827)

©2020 Hamedani AG et al. *JAMA Network Open.*

eFigure. Flowchart of inclusion and exclusion criteria for analyses of ONSF vs. CSF shunting for PTCS, NIS 2010-2016

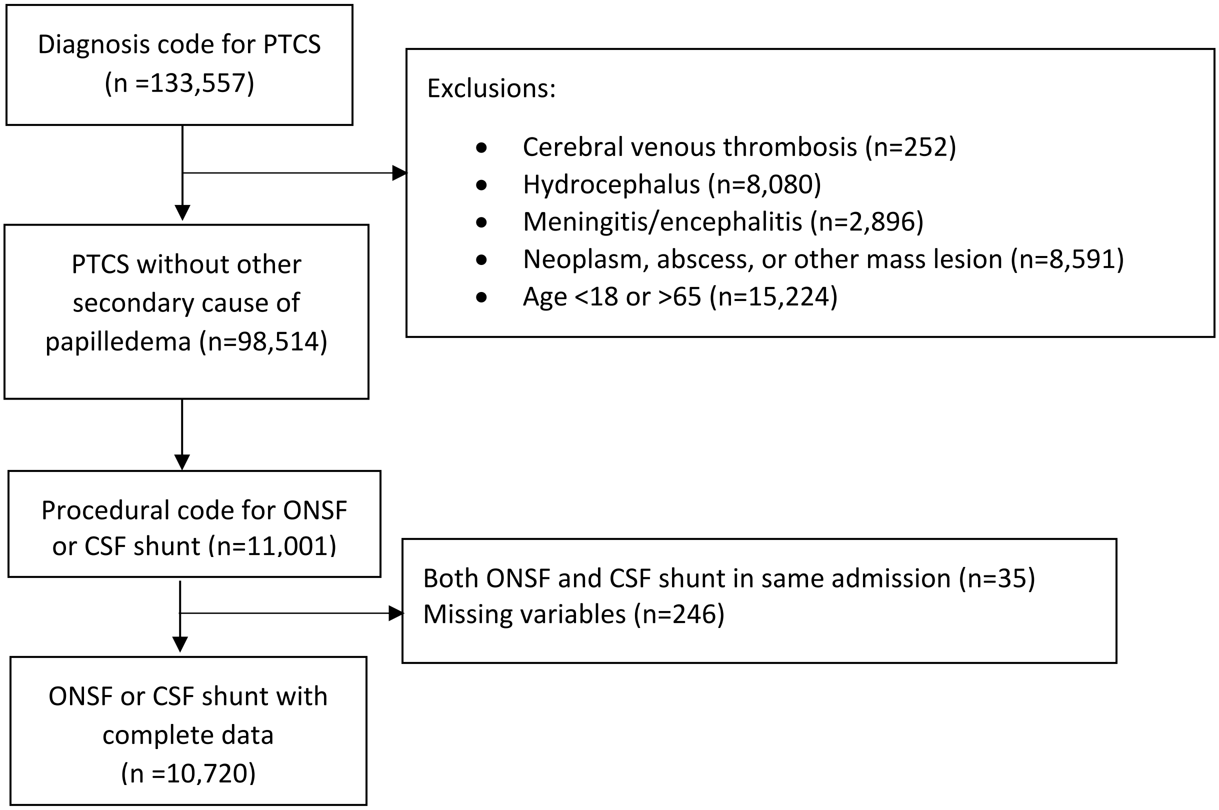

©2020 Hamedani AG et al. *JAMA Network Open.*
